# Development of a Preventive Health Screening Procedure Enabling Supportive Service Planning for Home-Dwelling Older Adults (PORI75): Protocol for an Action Research Study

**DOI:** 10.2196/48753

**Published:** 2023-10-03

**Authors:** Jonna Carita Kanninen, Anu Holm, Anna-Liisa Koivisto, Pauliina Hietasalo, Anna-Maija Heikkilä, Susanna Kunvik, Jussi Bergman, Marja Airaksinen, Juha Puustinen

**Affiliations:** 1 Faculty of Technology Satakunta University of Applied Sciences Pori Finland; 2 Clinical Pharmacy Group, Division of Pharmacology and Pharmacotherapy Faculty of Pharmacy University of Helsinki Helsinki Finland; 3 Satasairaala Central Hospital Pori Finland; 4 Satakunta Wellbeing County Pori Finland; 5 Faculty of Health and Welfare Satakunta University of Applied Sciences Pori Finland; 6 Unit of Neurology Satasairaala Central Hospital Satakunta Wellbeing County Pori Finland

**Keywords:** health screening, older adults, community health care, secondary use, Finland, screening, supportive service, clinical patient data, community, develop, primary care, self-assessment, pilot test, testing, planning, data collection

## Abstract

**Background:**

In Finland, at least 1 in 4 residents will be >75 years of age in 2030. The national aging policy has emphasized the need to improve supportive services to enable older people to live in their own homes for as long as possible.

**Objective:**

This study aimed to develop a preventive health screening procedure for home-dwelling older adults aged 75 years to enable the use of clinical patient data for purposes of strategic planning of supportive services in primary care.

**Methods:**

The action research method was applied to develop the health screening procedure with selected validated health measures in cooperation with the local practicing interprofessional health care teams from 10 primary care centers in the Social Security Center of Pori, Western Finland (99,485 residents, n=11,938, 12% of them >75 years). The selection of evidence-based validated health measures was based on the national guide to screen factors increasing fall risk and the national functioning measures database. The cut-off points of the selected health measures and laboratory tests were determined in consecutive consensus meetings with the local primary care physicians, with decisions based on internationally validated measures, national current care guidelines, and local policies in clinical practice.

**Results:**

The health screening procedure for 75-year-old residents comprised 30 measures divided into three categories: (1) validated self-assessments (9 measures), (2) nurse-conducted screenings (14 measures), and (3) laboratory tests (7 measures). The procedure development process comprised the following steps: (1) inventory and selection of the validated health measures and laboratory tests, (2) training of practical nurses to perform screenings for the segment of 75-year-old residents and to guide them to possible further medical actions, (3) creation of research data from clinical patient data for secondary use purposes, (4) secondary data analysis, and (5) consensus meeting after the pilot test of the health screening procedure for 75-year-old residents procedure in 2019 based on the experiences of health care professionals and collected research data.

**Conclusions:**

The developed preventive health screening procedure for 75-year-old residents enables the use of clinical patient data for purposes of strategic planning of supportive services in primary care if the potential bias by a low participation rate is controlled.

**International Registered Report Identifier (IRRID):**

DERR1-10.2196/48753

## Introduction

Between the years 2015 and 2050, the number of people aged >60 years will increase globally from 900 million to 2 billion [1]. In Finland, at least 1 in 4 residents will be >75 years of age in 2030 [2]. Health care systems should be realigned to meet the needs of growing older populations and be capable of providing person-centered integrated care that meets the needs of this population segment. Most health systems globally are ill-prepared to address the service needs of older people who often have multiple chronic conditions or geriatric syndromes.

Finland has one of the fastest-aging populations in the world [2]. Finnish aging policy and health and social policy have emphasized the need to enhance support services to enable older people to live in their own homes for as long as possible, even when daily home care support is regularly needed [2-4]. To enhance support for living at home, new effective preventive actions are needed to maintain the functional ability of older adults [2]. Early detection of diseases and efforts to prevent or attenuate the complications of chronic diseases may provide a cost-effective strategy for maintaining the health and functional ability of older adults, although conclusive evidence is still lacking [5-7]. Screening for the subclinical state of a disease has benefitted many asymptomatic patients [8] but applying preventive health screening to old people may be problematic for several reasons such as geriatric disorders with multiple risk factors, having less physiological reserve than younger adults, and having higher comorbidity rate often leading to polypharmacy. In general, older adults are often excluded from clinical trials influencing outcomes measured and reported for evidence synthesis and thus interpreted for medical interventions [8,9]. The abovementioned issues have contributed to the lack of comprehensive health screening procedures suitable for home-dwelling older adults and suitable to be used in clinical practice to guide preventive actions.

Our extensive literature searches did not find any studies reporting preventive comprehensive health screening tools or procedures specifically designed and validated for home-dwelling older adults. Existing age-specific health screening tools are disease or symptom specific (eg, cancer [10], mental disorders [11], frailty [12], cognitive impairment [13], falls [14], and nutritional status [15]) [10-21] and they primarily focus on community settings [17,22-26]. As health systems are becoming increasingly digitalized, screening tools should be suitable for structured and cumulative data recording in electronic patient databases, enabling data-driven management practices and the secondary use of social and health data in the future. The aim of this study was to develop a preventive health screening procedure for home-dwelling older adults aged 75 years to enable the use of clinical patient data for the strategic planning of supportive services in primary care.

## Methods

### Context of the Study

The Finnish health care and social services system is currently undergoing a major reform [27]. Since the beginning of 2023, these services have been organized by 22 well-being service counties [28,29]. According to the new Act on Organizing Social Welfare and Healthcare [30], the planning and implementation of publicly funded health care and social services in the well-being service counties should be guided by patient data aggregated in care processes. The Act on the Secondary Use of Health and Social Data [31] was enacted in 2019 and states how patient data can be used by service providers for organizing services, planning their equal availability, and optimizing cost-effectiveness [30].

This study was conducted in the Social Security Center of Pori, which was transferred to the well-being service county of Satakunta at the beginning of 2023 (1 of the 22 regional providers of social and health services in Finland) [28,29]. In the Satakunta Wellbeing Service County, institutional care for older residents has been significantly more common than in the rest of Finland. Therefore, the county made a strategic decision in 2019 to find new ways to remarkably reduce the proportion of older residents aged >75 years requiring expensive institutional care (reduction from 9% to 4.5% by 2024) [32]. The goal was for older residents to favor living at home for as long as possible by providing them with new community housing solutions, extending home care into round-the-clock services, and resourcing client-specific home care to support residents in poorer health. This study was started to support the strategic goal of the well-being service county by developing a preventive health screening procedure to identify home-dwelling older adults at risk of impaired health and functional ability.

According to the Finnish legislation, it is mandatory for the well-being service counties to use the Resident Assessment Instrument (RAI) assessment tools [33,34] in assessing the functional capacity of an older person if the person needs regularly provided social services to ensure sufficient care and support [35]. RAI is a comprehensive assessment tool specifically designed for the evaluation of the care needs of vulnerable populations [33,34]. The RAI assessment system consists of several assessment instruments designed for varied care contexts such as nursing home [36], home care [37] rehabilitation [38], palliative care [39], or acute care [40]. In contrast, a preventive health screening assesses home-dwelling older adults’ overall potential health risks and can be used for individuals regardless of their service needs.

This study included 10 public health care centers from 3 municipalities within the Satakunta Wellbeing County—Pori, Ulvila, and Merikarvia—located in Western Finland, with 99,485 inhabitants, of whom 12% (n=11,938) were ≥75 years in 2020 [41]. Pori Home Care Unit is divided into 3 service areas: North, West, and East. Each service area has 3-4 local public health centers. Every health center has a practical nurse (PN) who is trained in health screening. Health screenings are a publicly funded part of primary care services in Finland [42]. Since the beginning of 2023, well-being service counties have been funded mainly by the government and partly by collecting service fees from service users [28].

### Study Design and Method

This study applied the action research method to develop a preventive health screening procedure for home-dwelling older adults aged 75 years [43,44]. The action research method is suitable for identifying problems in clinical practice and developing potential solutions to improve the practice [43]. It involves researchers working with and for people rather than undertaking research on them [43]. The study design was developed in cooperation between the Satakunta University of Applied Sciences, the Social Security Center of Pori, and the University of Helsinki, Finland. Advanced-level nursing students from the Satakunta University of Applied Sciences were also involved in training PNs to conduct health screenings.

### Development Process of the Health Screening Procedure

The following were the starting points for the development of the health screening procedure: (1) the screening could be performed every year for a new segment of 75-year-old residents; (2) the health measures included should provide sufficient information about the health status of the home-dwelling residents in this age segment to plan for required health services and be suitable for structured and cumulative data recording enabling data-driven management practices; and (3) the health screening procedure should be dynamic, allowing changes in measures while maintaining comparability between annual cohorts to observe the possible changes in the health status of 75-year-old residents and their service needs over time.

**Figure 1:**
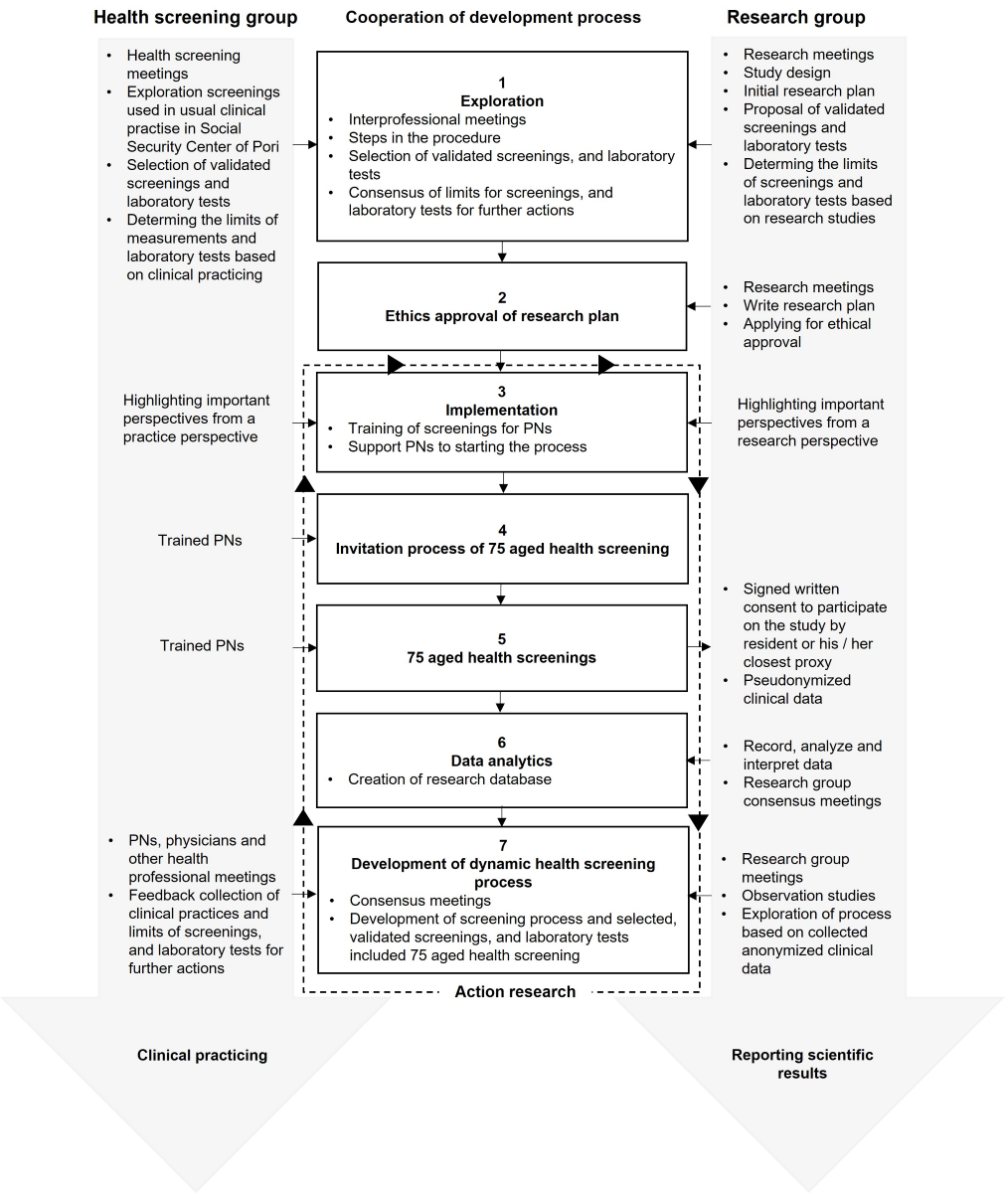
The development process of the preventive health screening procedure for home-dwelling older adults aged 75 years (PORI75) using the action research method [43,44]. PN: practical nurse.

The health screening procedure was developed in 7 steps and close cooperation between the health screening group—comprising the care team personnel from the Social Security Center of Pori—and the research group, comprising the researchers (Figure 1). The health screening group comprised all PNs, their managers (nurses), physicians, and other health care professionals involved in the 10 care centers of the 3 municipalities involved in the study. The research group comprised 3 clinically trained researchers: a physician, a pharmacist, and a clinical physicist.

### Ethical Considerations

This study was approved by the Ethics Committee of the Hospital District of Southwest Finland (ETMK 58/2019). The research permit was approved by the Social Security Center of Pori, Finland to conduct research at the health center. Informed consent was obtained from each client and their closest proxy before any study procedure was performed. This study was conducted in accordance with the principles of the Declaration of Helsinki.

### Inventory of Potential Health Measures to be Included in the Procedure (Step 1)

Meetings between the health screening group, which was familiar with health screenings in practice, and the research group conducting research played a crucial role in developing the health screening procedure (Figure 1). The first step was to make an inventory of potential health measures to be included in the procedure (research group). The inventory and selection of measures were based on evidence-based, validated, client-oriented measures covering the typical symptoms and conditions in older adults who may influence their functional ability to live at home. Our inventory found the national guide on preventing falls in older adults by the Finnish Institute for Health and Welfare as a useful evidence-based source of measures to be used in our study [45]. The guide presented a holistic approach to factors that have been associated with an increased risk of falls and that are recommended to be screened in older adults. Thus, we drew all the recommended health measures from the guide to form a preliminary set of measures for our health screening procedure.

In cases where a validated health measure recommended by the fall prevention guide [45] was not available in the guide, it was drawn from the National TOIMIA Functioning Measures Database, which is also maintained by the Finnish Institute for Health and Welfare [46]. The TOIMIA Functioning Measures Database is an open-access and free-of-charge database designed for experts and professionals to measure functioning in clinical practice and research. The database contains the basic descriptions of functioning measures, assessments of psychometric properties, and the feasibility of these measurement instruments for different purposes and guidelines and recommendations by experts concerning the measure of functioning in different situations and contexts. The guidelines and measures published in the database have been systematically evaluated by the TOIMIA network of experts with regard to their validity, reliability, and usability for different purposes.

**Figure 2:**
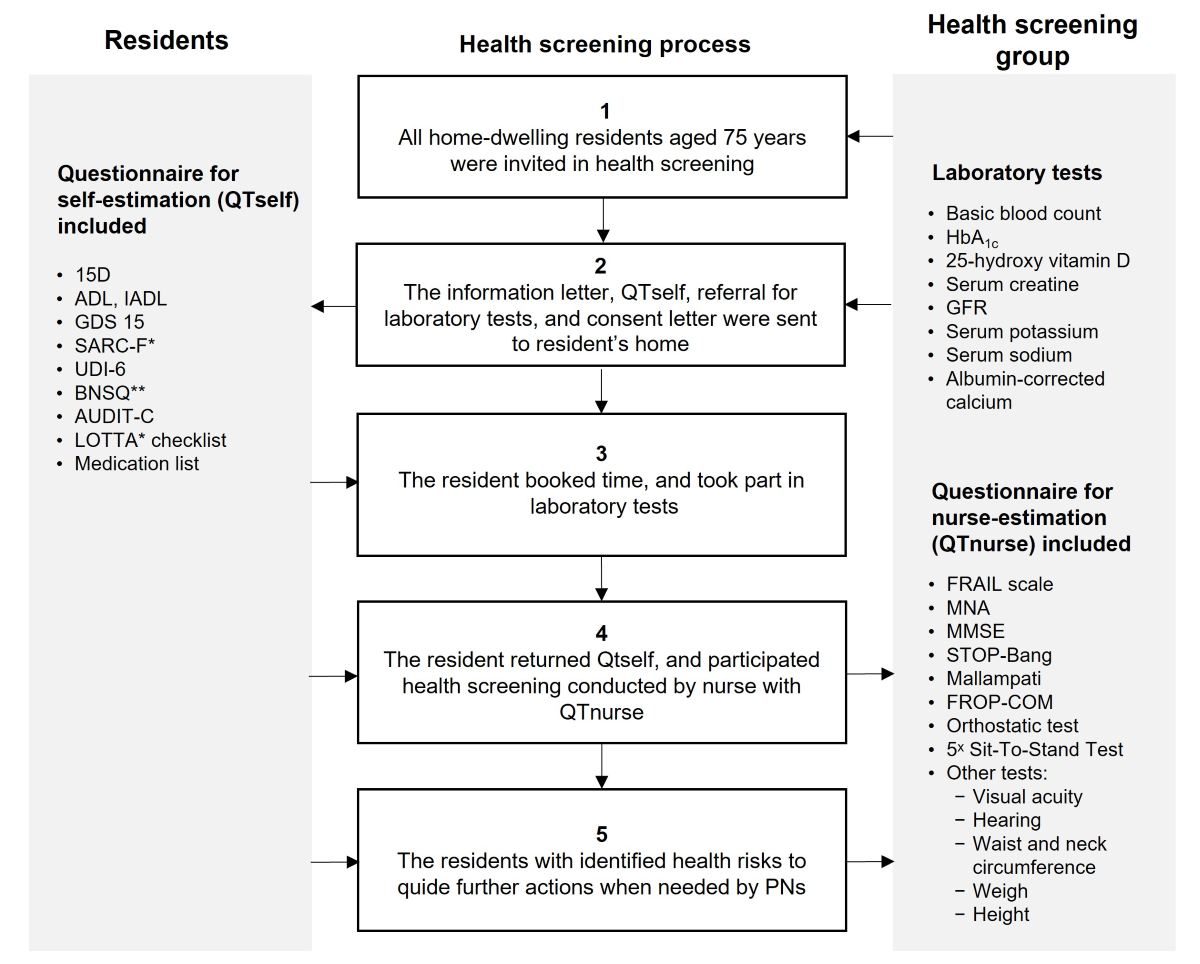
Description of the developed health screening procedure for 75-year-old residents (PORI75). * LOTTA checklist and SARC-F test were included in 2020. ADL: activities of daily living; AUDIT-C: Alcohol Use Disorders Identification Test—Consumption; BNSQ: Basic Nordic Sleep Questionnaire; FROP-Com: Falls Risk for Older People in the Community; GDS 15: Geriatric Depression Scale; GFR: glomerular filtration rate; HbA1c: hemoglobin A1c; IADL: Instrumental Activities of Daily Living; PN: practical nurse; QTnurse: questionnaire included screenings performed by a health care professional; QTself: questionnaire included screenings suitable for self-assessment; UDI-6: Urinary Distress Inventory; LOTTA: Checklist for Successful Medication Therapy (translated in English). ** The BNSQ questionnaire was included only in 2019, not in 2020.

When possible, the health measures used in routine clinical practice at the Social Security Center of Pori were selected for the health screening procedure to minimize additional work for PNs and inconvenience for residents (Figure 2). Laboratory tests were selected to screen for diseases and conditions prevalent in older adults according to the fall prevention guide [45] and to optimize drug treatment.

The cutoff points of the selected health measures and laboratory tests for possible further medical examination to treat the identified health problems were agreed upon in a consensus meeting with the local physicians (Multimedia Appendix 1 shows this in more detail). The criteria for the cut-off points were based on the categorization of internationally validated tests, national current care guidelines [47], and local policies. Consensus was reached when all the local physicians agreed on the cutoff points of the measures and tests in the meeting.

### Implementation of the Health Screening Procedure (Steps 2-7)

After consensus was reached between the research group and health screening group on the health measures and laboratory tests included in the procedure, named health screening procedure for 75-year-old residents (PORI75), a study protocol was developed to implement the procedure (Figure 1). The study protocol was evaluated by the Ethics Committee of the Hospital District of Southwest Finland (step 2).

For the implementation of the procedure in steps 3-7, all 75-year-old residents living at home were selected from 10 public health care centers in 3 municipalities within Satakunta Wellbeing Service County and were invited by a postal letter to participate in the health screening by a trained PN (Figure 1). The recruitment process was conducted between June 2019 and December 2020 and invited only those residents who turned 75 years old that year. All residents were given oral and written information about the study protocol prior to consenting. Written informed consent was obtained from all voluntary participants in person or with the help of their caregiver or closest proxy.

PNs were prepared to perform the health screenings for the consented residents of 75 years of age (Figure 1; step 3). During this preparatory phase, PNs were encouraged to comment on the health screening process to the research group to make it fit the usual clinical practice. PNs were trained to identify residents with elevated health risks in the health screening phase and to guide them to refer a health care professional or provider or visit a clinic for further medical examination to manage the problems when needed (Multimedia Appendix 1).

Step 5 was designed for implementing the health screenings using the health measures and tests included in the procedure in step 1 (Figure 1). The aim was to assess the current health status of home-dwelling 75-year-old residents by identifying potential ageing-specific issues for diseases prevalent in older adults as well as their medication-related risks.

In the data analysis step (step 6), the research group created a retrospective pseudonymized research database for the secondary use of the collected patient data (Figure 1). They analyzed the data and interpreted the obtained results to produce information on the health status of the residents and to identify the needs for health service use.

After the first segment of 75-year-old residents screened in 2019, the health screening group and research group conducted regular meetings to further develop the health screening process including the measures and tests used, and shared the preliminary results of the screening (step 7). The research group closely worked with PNs and physicians in consensus meetings to facilitate integration between the steps (steps 3-7) and the tasks of the health care professionals involved. The development of the dynamic health screening process as well as the appropriateness of the selected and validated screening measures is planned to be annually assessed in these consensus meetings (step 7). In addition, the health screening group will share the results of the screening with the steering committee to assess the quality and adequacy of available health services compared to the care and support needs of the home-dwelling older residents. This study was limited to describing the development process of the procedure, and the results of the screenings will be published in several different publications.

## Results

### Description of the Health Screening Procedure for 75-year-Old Home-Dwelling Residents

The health screening procedure for 75-year-old home-dwelling residents comprised 30 measures and tests divided into three categories: (1) validated self-assessments (9 measures), (2) nurse-conducted screenings (14 measures), and (3) laboratory tests (7 measures; Figure 2). Figure 2 presents the 5-stage process of conducting the measures and tests in practice.

The screenings are divided into 3 blocks, 2 of which are presented in 2 separate questionnaires (Figure 2). In the first block, the questionnaire is named as QTself (questionnaire included screenings suitable for self-assessment at home such as quality of life, functional capability, mood and depressive symptoms, the probability of sleep disorders, alcohol use, inability to urinate, and possible problems and risks in medication use; Table 1). In the second block, the questionnaire is named QTnurse (questionnaire included screenings performed by a health care professional) comprises screenings to be performed by a health care professional, that is, a trained PN. These screenings include frailty, nutritional status, cognition (memory and reasoning), sleep apnea, fall risk, lower body strength, orthostatism (difficulty in achieving stable blood pressure), and other tests such as those for identifying impaired vision and hearing (Table 2). The third block, the laboratory tests, includes the following tests such as basic blood count, long-term blood glucose, vitamin D, serum creatine, glomerulus filtration rate, serum sodium and potassium, and albumin-corrected calcium (Table 3).

**Table 1:** Health screening procedure for 75-year-old residents: the measures included in the self-assessment instrument (QTself questionnaire) are designed to be used by 75-year-old home-dwelling residents by themselves or with the help of their caregiver or closest proxy.

Test and health measure	Definition and description
*ADL, IADL* ^a,b,c,d^	ADL [48] is the scale of assessment of daily activities, where the amount of help needed in basic daily activities is scored, and the total of the points describes the amount of help needed by others. IADL [49] describes the need for assistance in instrumental daily activities.
*AUDIT-C* ^a,b,e^	Alcohol Use Disorder Identification Test (AUDIT) [50] is a screening instrument for hazardous and harmful alcohol consumption. (AUDIT-C) [51] questions have been previously validated as a 3-item screening tool for alcohol misuse.
*GDS 15* ^a,b,f^	GDS 15 [52] is a validated self-rating depression screening scale for older adults.
BNSQ^g,h,i^	BNSQ [53] has been used widely in a variety of studies and has proven to be a valid tool for measuring subjective sleep complaints.
15D^i,j^	15D [54] is a generic, comprehensive, 15-dimensional, standardized, and self-administered measure of health-related quality of life that can be used both as a profile and single index score measure.
SARC-F^i,k,l^	The SARC-F [55] questionnaire is a rapid diagnostic test for sarcopenia and covers the following components: Strength, Assistance with walking, Rise from a chair, Climb stairs and Falls.
UDI-6^i,m^	UDI-6 [56,57] is for assessing the life impact and symptom distress of urinary incontinence and related conditions for both men and women.
LOTTA checklist^i,l,n^	The LOTTA checklist is a validated self-assessment tool [58] for older adults aged >65 years to identify medication-related risks. The checklist aims to (1) ensure that the medication is being taken as directed, (2) encourage older adults to maintain an up-to-date medication list, and (3) monitor the therapeutic effects of their medication.
Medication list^i^	A medication list comprises the medicines currently in use and is filled by the older adult themselves or by the closest proxy at home.

^a^A measure included in the fall prevention guide [45].

^b^Measures given in italics are already in use at the Social Security Center of Pori before the PORI75 procedure was established.

^c^ADL: activities of daily living.

^d^IADL: Instrumental Activities of Daily Living.

^e^AUDIT-C: Alcohol Use Disorders Identification Test—Consumption.

^f^GDS 15: Geriatric Depression Scale.

^g^BNSQ: Basic Nordic Sleep Questionnaire.

^h^BNSQ was included only in 2019 and was excluded based on the experiences of its use in 2019.

^i^A measure recommended to be screened in older adults by the fall prevention guide [45] but the measure was retrieved from the National TOIMIA Functioning Measures Database [46].

^j^15D: comprehensive health-related quality of life instrument

^k^SARC-F: Strength, Assistance with walking, Rise from a chair, Climb stairs, and Falls.

^l^The LOTTA checklist and SARC-F test were included as new measures in 2020.

^m^UDI-6: Urinary Distress Inventory.

^n^LOTTA: Checklist for Successful Medication Therapy (translated in English).

**Table 2:** The health measures planned to be screened at the nurse’s appointment by trained PNs as part of the health screening procedure for 75-year-old residents (QTnurse questionnaire).

Test and health measure	Definition and description
*FROP-COM* ^a,b,c^	FROP-Com [59] are tools used in the community for the assessment or screening of falls risk.
*MMSE* ^a,d^	MMSE [60] is a test of cognitive function among older adults.
*MNA* ^a,e^	MNA [61] is a simple tool useful in clinical practice to measure nutritional status in older persons.
*Orthostatic test* ^a^	The orthostatic test [62] is used to determine how the position of the body affects the heart rate and blood pressure. Orthostatic hypotension is defined as a decrease in systolic blood pressure of at least 20 mm Hg or in diastolic blood pressure of 10 mm Hg when assuming an upright position.
5×sit-to-stand test^a^ (Chair stand, 5 times)	The 5 times sit to stand test (5×sit-to-stand test) [63,64] is used to assess the functional lower extremity strength, transitional movements, balance, and fall risk in older adults.
FRAIL scale^f,g^	The FRAIL scale [65] is a screening tool for clinicians to identify frail persons at risk of developing disability as well as experiencing a decline in health functioning and mortality.
STOP-Bang, Mallampati^f,h,i^	The STOP-Bang [66,67] questionnaire is used to screen patients for obstructive sleep apnea. The Mallampati [68] score is used to identify patients at risk for difficult tracheal intubation and evaluate obstructive sleep apnea.
Other tests^b^ Visual acuity^a^ (random E test) Hearing^a^ (tympanometry) Weight^a^ Height^a^ Waist circumference^f^ Neck circumference^f^	Clients are also examined, and the values for disease identification and those necessary for the study are measured.

^a^A measure included in the fall prevention guide [45].

^b^Measures given in italics are already used at the Social Security Center of Pori.

^c^FROP-Com: Falls Risk for Older People in the Community.

^d^MMSE: Mini-Mental State Examination.

^e^MNA: Mini Nutritional Assessment.

^f^A measure recommended to be screened in older adults by the fall prevention guide [45] but the measure retrieved from the National TOIMIA Functioning Measures Database [46].

^g^FRAIL: simple frailty questionnaire; fatigue, resistance, aerobic capacity, illnesses and loss of weight.

^h^The measure is not specific and sensitive for older adults.

^i^STOP-Bang: Score for obstructive sleep apnea, Snoring, Tired, Observed, Pressure, Body Mass Index.

All chosen measures are specific and sensitive for older adults, except the quality of life measure (Comprehensive Health-Related Quality of Life Instrument [15D]) [54] and 2 measures for sleeping disorders (Score for obstructive sleep apnea, Snoring, Tired, Observed, Pressure, Body Mass Index [STOP-Bang] [66,67] and Mallampati [68]). The 15D is a comprehensive health-related quality of life instrument for adults but is not specifically designed for older adults [54]. In the same way, STOP-BANG [66,67] and Mallampati [68] are used to evaluate obstructive sleep apnea in adults but not particularly in older adults.

**Table 3:** Laboratory tests are included in the PORI75 health screening procedure for 75-year-old home-dwelling residents to screen for diseases prevalent in older adults.

Laboratory tests	Definition and description
25-hydroxy vitamin D^a^	The measurement of serum vitamin level is generally recognized as the clinical standard for the evaluation of vitamin D status.
Albumin-corrected calcium^b^	Total plasma calcium is bound to protein, with albumin being the most abundant binding protein. Albumin-corrected calcium is used as a measure of calcium balance.
Basic blood count^b^	The basic blood count is an important basic laboratory examination that indicates various ways to analyze the condition of the body.
Hemoglobin A_1c_^b^	Glycated hemoglobin levels are a measure of average sugar levels in the blood and are commonly used in relation to diabetes.
Serum creatinine glomerular filtration rate^c^	A blood creatinine examination is a part of a routine health examination for recognizing kidney damage or the risk of developing kidney disease. Glomerular filtration rate is the measurement used to determine kidney function.
Serum potassium^c^	Serum potassium is a measure of average potassium levels in the blood.
Serum sodium^c^	Serum sodium is a measure of average sodium levels in the blood.

^a^A measure included in the fall prevention guide [45].

^b^A measure recommended to be screened in older adults by the fall prevention guide [45].

^c^A measure recommended to optimize drug treatment.

### Performing the Pilot PORI75 Health Screenings in 2019

All residents (n=1104) who turned 75 years old in 2019 and lived at home were sent an invitation letter to participate in the PORI75 health screening (Figure 2, stage 1). Of the invited residents, the participation rate for health screening was 37% (n=415). Among these 415 participants, 407 (98%) gave written consent to be included in the study.

The invitation letter contained a reserved time for the PN’s appointment, a request to fill in the QTself questionnaire before the appointment, instructions to book a slot for laboratory tests (Table 3), and an informed consent letter for the study participation (Figure 2, stage 2). The residents were recommended to book an appointment for the laboratory tests no later than 5 days before the PN’s appointment (Figure 2, stage 3). They were also asked to return the filled QTself form at the PN’s appointment when the PN conducted the health screenings according to the QTnurse form and reconciled the medication list (Figure 2, stage 4). Residents signed the written informed consent during the PN’s appointment if they were willing to participate in the study. An appointment took about 2 hours including screening, reconciling the medication list, and recording results in the electronic patient information system. If a PN identified potential health risks based on the predetermined health risk cut-off points (Multimedia Appendix 1), they advised the resident to contact a health care provider for further medical examination to manage the observed health risks (Figure 2, stage 5).

### Experiences From the First Pilot-Year Screenings in 2019

The consensus meeting after the pilot screenings in 2019 considered the content of the PORI75 procedure to be appropriate and applicable to a new segment of 75-year-old residents in 2020. The only changes made to the measures were the removal of the Basic Nordic Sleep Questionnaire [53] for measuring sleep disorders from the procedure. This is because the Basic Nordic Sleep Questionnaire [53] lacked an easily applicable and validated scoring method, thus making it difficult for PNs to identify residents at risk. In addition, the following 2 new measures were added to the health screening: a tool (Strength, Assistance with walking, Rise from a chair, Climb stairs, and Falls) for identifying sarcopenia in older adults [55] and a checklist (LOTTA) for assessing medication-related risks and problems [58]. Strength, Assistance with walking, Rise from a chair, Climb stairs, and Falls measure [55] was chosen because sarcopenia may impede daily activities and increase the rate of complications in older persons [69]. The validated LOTTA checklist was nationally launched in Finland in 2020 by the Finnish Medicines Agency [58]. This self-assessment tool for adults aged >65 years aims to identify the potentially harmful risks and problems in their medication use.

## Discussion

### Principal Findings

The PORI75 preventive health screening procedure was successfully developed and piloted with 75-year-old home-dwelling residents. The procedure includes a coordinated set of health measures covering the most common age-related diseases and other conditions that may affect older adults’ functionality and survival at home. The established questionnaires of health measures and laboratory tests enable systematic and structured patient data recording and management, with potential for secondary use in planning preventive services for older adults according to their needs. This aligns with the ongoing social and health care reform goals in Finland [70]. The developed health screening procedure is dynamic and can be expanded and annually supplemented with new health screening data from 75-year-old residents. At the moment, the database includes data from the years 2019-2022. If the number of measures is wanted to be increased, modern pervasive computing technology with sensors could provide new additions enabling continuous monitoring of selected health functions in home-dwelling older adults [16,17,71].

The final health screening procedure consists of quite a high number of validated measures (n=30 measures), some of which are extensive and time-consuming to apply (eg, quality of life [54] and functional ability [63,64] measures). This increases the workload of the Social Security Center personnel, especially the PNs who perform the measures. However, the majority of the measures (22/30, 73%) were already in use at the Social Security Center before the PORI75 procedure was established. Although these health measures were used in different health care wards and contexts, systematic preventive screening was missing. Of the existing measures, 10 measures were included in the QTnurse questionnaire and 7 in laboratory tests of the PORI75 procedure.

Delegating the task of self-assessment for some measures to the older residents themselves proved to be a feasible practice (9 measures, of which 5 were already applied at the Social Security Center before PORI75). Based on the pilot experiences, 75-year-old home-dwelling residents were able to actively participate in health screening by answering the QTself questionnaire for the chosen measures. As all the residents were interviewed by a PN during their health screening appointment, PNs were able to confirm whether all measures of QTself questionnaire had been completed and what problems the residents may have experienced in filling in their personal information. Thus, we can assume that all residents who consented to fill the QTself questionnaire did so. Unfortunately, the PNs did not keep records of the difficulties the residents may have experienced in completing the QTself questionnaire, so we cannot report these findings. Difficulty in self-completing the questionnaires could have been one of the reasons for not participating in the health screening. These findings suggest the need for making a more detailed analysis of health screening participants and nonparticipants in the future.

The participation rate in the health screening was not very high (n=415, 37%), even though the health screening was provided free of charge for all 75-year-old home-dwelling residents. If the health screening for 75-year-old residents will become a routine practice, it is important to find new strategies to increase the participation rate. Otherwise, the procedure will not reach its primary goal of helping older residents to live at home for as long as possible and lowering the proportion of older residents living in assisted living facilities and nursing homes.

Another consequence of the low participation rate can be biased on estimates for planning social and health services needed for geriatric care. The bias in the allocation of resources can be aggravated by the assumption that many nonparticipants may have had a poorer health status and functional ability than the participants. This will lead to underestimating the resources needed for geriatric care. Therefore, it is important in future studies to investigate the characteristics of health screening participants and nonparticipants to estimate the bias caused by the participation rate. Based on this analysis, a suitable correction should be used in data analysis to consider this potential source of bias that can reflect on health services planning and allocation of resources in the well-being services county. It is also important to understand other complications encountered in the process and to focus on them in future studies. A potential option for increasing the participation rate could be to conduct health screening in connection with emergency department visits. It is important to note that in any case, the health screening is entirely voluntary, and it is everyone's right to choose whether they want to participate.

In Finland, 75-year-old residents or any other segment of older adults do not routinely have PN’s appointments and the related opportunities for preventive health counseling such as those presented here. Systematic health checks are routine in most countries [72] but are more typically focused on specific diseases or symptoms [10-21] or medication management [73]. The PORI75 is a unique and comprehensive health screening procedure for home-dwelling older adults where the effectiveness and cost-effectiveness should be evaluated in future studies when we have gathered sufficient data for statistical analyses. In forthcoming studies, we will evaluate the effectiveness of the health screening by comparing emergency department visits before and after the health screening for both participants and nonparticipants. If the outcomes are positive, the procedure has the potential to be implemented in other well-being services counties, or even nationally in Finland and beyond. As the measures and tests were chosen by the local health screening group based on proposals from the interprofessional research group, consensus meetings with an extended representation should be carried out to confirm the consensus findings.

The PORI75 health screening procedure is still under development. The procedure has been pilot-tested and used in clinical practice at the Social Security Center of Pori as presented in this study. The development continues towards a digital data lake and a template facilitating the use of the data in data-driven management in the regional well-being services county.

### Conclusions

The developed preventive PORI75 enables the use of clinical patient data for purposes of strategic planning of supportive services in primary care if the potential bias by a low participation rate is controlled.

## Appendix

Multimedia Appendix 1 The cut-off points of screenings and laboratory tests for further actions.

## Abbreviations

15D: Comprehensive Health-Related Quality of Life Instrument
PN: practical nurse
PORI75: health screening procedure for 75-year-old residents
QTself: questionnaire included screenings suitable for self-assessment
QTnurse: questionnaire included screenings performed by a healthcare professional
RAI: Resident Assessment Instrument
STOP-Bang: Score for obstructive sleep apnea, Snoring, Tired, Observed, Pressure, Body Mass Index

## Data Availability

The data that support the findings of this study are available from the Social Security Center of Pori but restrictions apply to the availability of these data, which were used under license for the current study and are therefore not publicly available. Data are, however, available from the patient document archive of Pori Town (arkisto.ter@porinperusturva.fi) based on the Act on the Secondary Use of Health and Social Data upon reasonable request.
