# Supplementary material for: Development of a Preventive Health Screening Procedure Enabling Supportive Service Planning for Home-Dwelling Older Adults (PORI75): Protocol for an Action Research Study
Source: JMIR Res Protoc. 2023 Oct 3;12:e48753. doi: 10.2196/48753 (PMC10582811; doi:10.2196/48753)
Supplement: Multimedia Appendix 1 [file resprot_v12i1e48753_app1.docx]

**Multimedia Appendix1**

The cut off points of screenings and laboratory tests for further actions.

| **Screenings of QTself** | **Reference value** | **Interpretation** | **Guidance to a healthcare professional/clinic** |
| --- | --- | --- | --- |
| ADL (32) and IADL (33) | ADL:  Score ranges from 0 (patient very dependent) to 6 (patient independent):   - 6 indicates full function - 4 indicates moderate impairment - 2 or less indicates severe functional impairment   IADL:  Score ranges from 0 (low function, dependent) to 8 (high function, independent):   - Women: 0 (low function, dependent) to 8 (high function, independent) - Men: 0 through 5 | The nurse’s own assessment | If necessary, guidance for to the service instructor |
| AUDIT-C (37, 38) | Score ranges 0–12 (scores of 0 reflect no alcohol use)   - Men ≥4 - Women ≥3 | If doses ≥ 3 at once or 7 doses per week  Alcoholism | A nurse  A-clinic |
| GDS 15 (34, 52) | Score 1 point of the following 15 answers:  Of the 10 indicated the presence of depression when answered positively, while the rest (question numbers 1, 5, 7, 11, 13) indicated depression when answered negatively:   - 0–4 are considered normal, depending on age, education, and complaints - 5–8 indicate mild depression - 9–11 indicate moderate depression - 12–15 indicate severe depression | If points ≥ 6 | If the first time, guidance for to nurse specialized in mood disorders  If depression diagnosed, nurse recommends contacting own psychiatric |
| 15D (28) | Answer scale: 1-5  (Instrument for questionnaire: The minimum important changes (MICs) and minimum important differences (MIDs) of 15D scores are ±0.015) | If points ≥ 3 | A nurse discusses with patient about situation |
| SARC-F (6) | Score ranges 0-10. Answer scale 0-2 in every question.   - A score ≥ 4 defines sarcopenia. | If points ≥ 4 and  5x Sit-To-Stand Test is ≥ 15 seconds duration | A physiotherapist |
| UDI-6 (35, 36) | Score range 0-18 points. Answer scale 0-3 in every question.  Higher scores indicate more symptom distress | If points 2 or 3 in some question and a patient experiences significance harm | A nurse discusses with patient about situation and for call queue to physician if needed |
| LOTTA checklist (40) | - | If regular chronic disease medication monitoring, follow-up visits or home measurements lacking  If identified from list (question 4.) any side effect symptoms of drugs | Reference to pharmacist |
| Medication list | - | If not filled in correctly | Medication reconciliation in nurse appointment if needed |
| **Screenings of QTnurse** | **Reference value** | **Interpretation** | **Guidance to a healthcare professional/clinic** |
| FROP-COM (43) | Score range of 0 to 9 (answer scale 0–3), grading of falls risk:   - 0–3 Low risk - 4–7 Medium riski - 8–9 High risk | If points 6 or 7  If points 8 or 9 | A physiotherapist  A physiotherapist specialized in elderly |
| MMSE (42) | The maximum MMSE score is 30 points and represents:   - 25–30 points: normal cognition - 21–24 points: mild dementia - 10–20 points: moderate dementia   9 points or lower: severe dementia | If points ≤ 27 | A nurse specialized in memory disease |
| MNA (41) | The sum of the MNA score distinguishes between elderly patients with:  1) normal nutritional status,  MNA ≥ 24  2) at risk of malnutrition,  MNA between 17–23.5  3) malnourished,  MNA < 17 | If points ≤ 17 or  if BMI < 23 and weight loss > 3 kg in the last 3 months  If points 17–23,5  If points ≥ 24 | A physician and dietician  A nurse’s assessment of the need for physician´s/ dietician´s guidance and guide to pay attention to diet  Guidance to pay attention to varied diet |
| Orthostatic test (44) | - Orthostatic hypotension is defined as a decrease in systolic blood pressure of 20 mmHg or a decrease in diastolic blood pressure of 10 mmHg within three minutes of standing when compared with blood pressure from the sitting or supine position | If systolic blood pressure in a hypertensive patient is under 130 mmHg  If the upper pressure drops to 20 mmHg or more, the lower pressure drops to 10 mmHg or more, and as a symptom of dizziness, weakness, or a fall | A physician’s consultation about the hypertensive medication  A physician |
| 5x Sit-To-Stand Test (45, 46)  (Chair stand, 5 times) | "Worse than the average": >12.6 s (70 – 79 years) | If more than a minute duration | A physiotherapist |
| FRAIL scale (7) | Scores ranges from 0–5 (i.e., 1 point for each component; 0=best to 5=worst) and represent   - frail (3–5) - pre-frail (1–2) - robust (0) health status | If 5 points  If 3–4 points | A geriatric outpatient clinic  A call queue to physician |
| STOP-Bang (29, 30) | Score ranges from 0 to 8:   - High risk of OSA: Yes 5 – 8 - Intermediate risk of OSA: Yes 3 – 4 - Low risk of OSA: Yes 0 – 2 | If points ≥ 6 | A physician  If the patient has already been diagnosed with sleep apnea, guidance to contact CPAP (Continuous Positive Airway Pressure) - giver |
| Mallampati (31) | Score, class 3 or 4 (category 1–4), is associated with more difficult intubation as well as a higher incidence of sleep apnea | If STOP-Bang is normal (< 3) and Mallampati is 4 | No action needed  A nurse records the findings in the patient information system |
| Other tests   - Visual acuity (random E test) - Hearing (tympanometry) - Weight - Height - Waist circumference - Neck circumference | - | If > 2 years since visiting the optician  If tympanometry deterioration (hearing) | An optician  Nurse performs hearing test |
| **Laboratory test** | **Reference value(53)** | **Interpretation** | **Guidance to a healthcare professional/clinic** |
| 25-hydroxy  vitamin D | < 50 nmol/l = Deficiency  > 375 nmol/l = Toxic concentration | If < 50 nmol/l and not use D-vitamin supplementation  If new finding:  >250 nmol/l | A nurse recommends using 20 µg vitamin D supplements throughout year and reserves new time for laboratory test.  A nurse recommends reducing 25 % vitamin D dose and reserves a new time for laboratory test for 3 months |
| Albumin-corrected calcium | 2.15-2.51 mmol/l | If new finding:  <2 mmol/l  >2.7 mmol/l | A physician |
| Basic blood count | Men: < 134 g/l or > 167 g/l  Women: < 117 g/l or > 155 g/l | If new finding:  Men: < 125 g/l or > 180 g/l  Women: < 115 g/l or > 170 g/l | The nurse’s own assessment of urgency |
| Hemoglobin A1c (HbA1c) | 20–42 mmol/mol | If new finding:  ≥ 48 mmol/mol | A physician |
| Serum creatinine  GFR | ≥70 aged, >59 ml/min/1.73 m^2^ | If new finding:  <50 ml/min/1.73m^2^ | A physician if renal impairment not known |
| Serum potassium | 3.3-4.9 mmol/l | If new finding:  <3 mmol/l  >5.5 mmol/l | A physician |
| Serum sodium | 137-145 mmol/l | If new finding:  <130 mmol/l  >150 mmol/l | A pharmacist for medication review and after that to physician |

*OSA: Obstructive sleep apnea
